# La cardiopatía congénita del adulto: un desafío de salud del presente y del futuro

**DOI:** 10.47487/apcyccv.v1i3.73

**Published:** 2020-09-30

**Authors:** Víctor Robles-Velarde

**Affiliations:** 1 Servicio de Cirugía Cardiovascular de Adultos - Instituto Nacional Cardiovascular INCOR. Lima, Perú. Servicio de Cirugía Cardiovascular de Adultos - Instituto Nacional Cardiovascular INCOR Lima Perú

## Introducción

El estudio y el tratamiento de las cardiopatías congénitas del adulto tiene sus orígenes desde que se advirtió que la supervivencia de los niños nacidos con esta patología aumentaba gracias a los progresos en su diagnóstico y tratamiento. En 1947, Helen B. Taussig publica el libro *Congenital Malformation of The Heart* iniciando el estudio sistematizado de las cardiopatías congénitas. Años antes, en 1938, Robert E. Gross realiza la primera ligadura del conducto arterioso permeable y, en 1944, Helen B. Taussig y Alfred Blalock efectúan en una niña cianótica con tetralogía de Fallot, la primera fístula sistémico pulmonar logrando la mejoría clínica de la paciente. Luego, en 1953, se utilizó por primera vez la bomba corazón-pulmón de Gibbon para la reparación bajo visión directa de una comunicación interauricular, comenzando así la corrección quirúrgica no solo de los defectos congénitos del corazón, sino de toda la patología cardiovascular.

Por todo lo descrito y ante la aparición de una nueva población de pacientes, en 1984 se funda la International Society for Adult Congenital Heart Disease (ISACHD), una organización sin fines de lucro para promover, mantener y buscar la excelencia en el cuidado de adultos con enfermedad cardíaca congénita en todo el mundo. El año 2001, el reporte de la American Heart Association, estimó que el 85% de los niños que nacen con una cardiopatía alcanzan la vida adulta y, por primera vez, el número de adultos con cardiopatías congénitas supera al de los niños ^1^. El 2012, el *American Board of Medical Specialties* aprobó el establecimiento de una nueva subespecialidad con necesidad de certificación: especialista en Cardiopatías Congénitas del Adulto.

## Una población creciente

El desarrollo de las nuevas técnicas de diagnóstico, de nuevos métodos de intervención y de tratamiento quirúrgico han prolongado la supervivencia de los niños que nacen con una cardiopatía congénita (CC) ^1^^-^^3^, llegando a alcanzar edades que no eran posibles anteriormente. La supervivencia de los pacientes con CC depende de qué tan complejo es el defecto, cuándo se diagnostica y cómo es tratado. Con la mejora del tratamiento de las CC se ha originado una población de adolescentes y adultos que alcanza mejoría de la clase funcional, de la calidad de vida y del pronóstico. Otro grupo de pacientes con CC no reciben ningún tratamiento, o no son diagnosticados en etapas tempranas de la vida, llegando a la adultez presentando sintomatología de alteraciones cardiovasculares propias de la historia natural de la enfermedad.

Por lo tanto, los pacientes adultos con CC son aquellos pacientes que fueron sometidos a algún tipo de tratamiento en la infancia como intervencionismo o cirugía y aquellos supervivientes naturales de su patología. Muchos de estos pacientes que sobreviven, presentan lesiones residuales, secuelas, complicaciones que pueden evolucionar durante la vida adulta y presentarse como alteraciones eléctricas, enfermedades valvulares, cortocircuitos persistentes, disfunción miocárdica, lesiones vasculares, alteración de los materiales protésicos implantados, infecciones como endocarditis, alteraciones tromboembólicas, alteraciones extracardiacas con afección a diversos órganos y sistemas, alteraciones psicológicas y muerte prematura **(**Tabla 1**)**^4^^,^^5^.

**Tabla 1:** Clasificación de los residuos, secuelas y complicaciones de las cardiopatías congénitas

Alteraciones electrofisiológicas	Cambios electrofisiológicos permanentes
	Arritmias y defectos de la conducción
Alteraciones valvulares	Malformaciones intrínsecas de las válvulas
	Secuelas de intervenciones anteriores
	Efectos hemodinámicos sobre válvulas normales
Cortocircuitos persistentes	Residuos no corregidos
	Secuelas de procedimientos terapéuticos
Disfunción miocárdica
	Alteraciones estructurales
	Hipertrofia y remodelamiento
	Isquemia perioperatoria
Alteraciones vasculares
	Estenosis congénitas y adquiridas
	Hipertensión pulmonar o sistémica
	Aneurisma, disección o rotura
Alteraciones de materiales protésicos	Parches, válvulas, conductos
Complicaciones infecciosas
	Endocarditis
	Vasos arteriales o fístulas
	Estructuras extravasculares
Fenómenos tromboembólicos
	Trombosis intravascular
	Tromboembolia pulmonar o sistémica
Alteraciones extravasculares
	Desarrollo psíquico y físico
	Órganos de los sentidos
	Estructura osteomuscular
	Sistema nervioso central
	Dentición
	Otros órganos y sistemas

Fuente: Cardiopatías congénitas del adulto: residuos, secuelas y complicaciones de las cardiopatías congénitas operadas en la infancia. José María Oliver Ruiz. Rev Esp Cardiol 2003;56(1):73-88.

## Población afectada

El informe de la 32.ª Conferencia de Bethesda el año 2001, estimó que había alrededor de 2800 adultos con cardiopatías congénitas por cada millón de habitantes, más de la mitad de ellos con defecto de complejidad moderada o alta ^6^. Se estima que aproximadamente 1 millón de niños y 1,4 millones de adultos estadounidenses viven con una CC y este número está previsto que continúe en ascenso a un ritmo de 5% por año ^1^^,^^7^. Se estima que el número de adultos con cardiopatías congénitas que viven actualmente en Europa sea de 1 millón de pacientes ^8^.

Diversos trabajos han establecido que la incidencia de las CC es de aproximadamente 0,8%, con un rango que va de 0,4 a 1,2% ^9^^-^^11^, las variaciones dependen del momento del estudio, de la población y de los métodos diagnósticos. Dentro de los tipos, las acianóticas son las más frecuentes, representando un 83% de todas las cardiopatías congénitas, y las cianóticas un 17% ^12^. En el Perú tenemos pocos trabajos sobre la incidencia de CC, en el estudio de Olortegui y Adrianzen del año 2007, calcula una incidencia de 8 por 1000 nacidos ^12^. Según el censo de población del Perú del año 2017 ^13^, nacieron alrededor de 600 000 niños, entonces aproximadamente nacen en el Perú alrededor de 4000 niños por año con alguna cardiopatía que va a requerir algún tipo de atención médica y muchos de ellos llegarán a la edad adulta con necesidades de atención médica especializada. En el Perú se desconoce el número de pacientes adultos que son portadores de alguna CC, si nos guiamos por las estimaciones de la 32.ª Conferencia de Bethesda, debe existir casi 90 000 adultos portadores de esta patología.

## Desafíos en la atención del paciente adulto con cardiopatía congénita

Con el trascurso de los años tenemos cada vez mayor incremento de pacientes adultos afectados con CC; por lo que hay que considerar: ¿qué necesidades nuevas se están produciendo?, ¿cómo se deben planificar estos nuevos requerimientos?, ¿cómo encontrar soluciones a esta nueva problemática de salud? Para conocer el número de pacientes con cardiopatía congénita que llegará a la edad adulta en los próximos años, se debe conocer primero el número de niños que nacen con una CC en nuestra población; luego, determinar el número de pacientes sometidos a algún tipo de tratamiento y sus niveles de supervivencia. Todo ello permitirá determinar las necesidades de recursos y su distribución en los diferentes niveles de atención de la salud.

La importancia de este grupo poblacional se refleja en la publicación de guías de práctica clínica que se van actualizando periódicamente ^14^^-^^17^, reconociendo los avances en el cuidado de la creciente población de adultos con CC. Hoy en día existen pocas instituciones dedicadas a la atención del adulto con CC; y, la enseñanza de esta patología, como parte de la preparación del cardiólogo de adultos, es aún escasa en la mayoría de los centros cardiológicos.

## Necesidad de recursos

**Figura 1:**
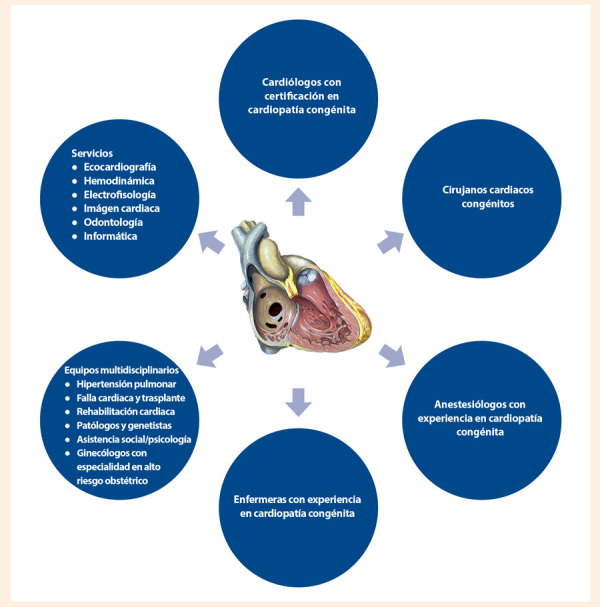
Equipo multidisciplinario en el manejo del adulto con cardiopatía congénita

Hay una creciente demanda de recursos de salud por parte de este grupo de pacientes, que se traduce en mayor número de consultas médicas, de hospitalizaciones, de procedimientos hemodinámicos, de cirugías cardiacas y otros ^18^^,^^19^. Son pacientes que, en ocasiones, han sido sometidos a múltiples cirugías, múltiples intervenciones, hospitalizaciones frecuentes, con afecciones de otros órganos y sistemas; lo que requiere el trabajo conjunto de múltiples especialidades en forma sincronizada **(**Figura 1**)**.

En concordancia con los desafíos presentes y futuros, en el Instituto Nacional Cardiovascular (INCOR-EsSalud) de Lima-Perú (centro de referencia nacional), se creó el año 2019 el Comité Multidisciplinario Para el Manejo de la Patología Cardiaca Congénita del Adulto; y, entre septiembre del 2010 a julio del 2020 se han sometido a tratamiento quirúrgico más de 500 pacientes adultos con CC de diversa complejidad (que incluyen cinco sometidos a trasplante cardiaco), cifra aún insuficiente dada el aumento creciente de esta población.

## Conclusiones

La CC del adulto es una entidad clínica que aumenta continuamente, con nuevas problemáticas y nuevos desafíos. Está presente en el paciente desde antes de nacer y la tendrá hasta el momento en que muera, por lo que su manejo debe involucrar a un equipo multidisciplinario para el manejo óptimo de este grupo especial de pacientes; por ello es importante la creación de centros de referencia especializados.

Ha llegado el momento de preparar al sistema de salud para este grupo creciente de pacientes que requieren atención permanente, integral y altamente especializada. Lamentablemente, en la actualidad no existen suficientes especialistas ni centros de atención que estén capacitados para asumir este reto en nuestro país.
